# 

**Published:** 1867-09

**Authors:** 


### MEDICAL EXCHANGES (IN PART.)

BOSTON MEDICAL AND SURGICAL JOURNAL, $4 a year, published by David Clapp & Son, 334 Washington street, Boston, Mass. Now in its 75th volume.

BUFFALO MEDICAL AND SURGICAL JOURNAL, $3 a year, edited by Julius F. Miner, M. D., Surgeon to the Buffalo General Hospital. Address Medical and Surgical Journal, Buffalo, New York.

DENTAL COSMOS, a monthly record of dental science, devoted to the interests of the profession, edited by Drs. McQuellan & Zilgler, $2.50 a year, single Nos. 25c. Philadelphia, Pa., 528 Arch street; 658 Broadway, New York; 16 Tremont Row, Boston; 100 Randolph street, Chicago, Ill.

DENTAL REGISTER, Cincinnati, Ohio, published monthly for $3.00 a year, is always well edited and richly merits the patronage of the profession.

HERALD OF HEALTH, and Journal of Physical Culture, $2 a year, single Nos. 20c., published by Miller, Wood & Co., 13 and 15 Laight street, New York. W. Tweedie, 337 Strand, London.

HOMEOPATHIST AMERICAN, edited by James G. Hunt, M.D., $1.50 a year, in advance, conducted by an able corps of editors, Drs. Bowling, Eve, Jones and Bleckie.

MEDICAL EXAMINER, Chicago, Ill., edited by N. S. Davis, M. D., professor of the principles and practice of medicine and of Clinical Medicine, &c., &c., $3 a year.

MEDICAL AND SURGICAL PIONEER, Kansas City, Mo., being a monthly record of medicine and surgery; $4 a year.

MEDICAL JOURNAL, Chicago, Ill., vol. 24, $2 per annum, 169 South Dearborn street; address R. M. Lackey, M.D., P. O. box 2175, Chicago, Ill. Edited by Drs. Holmes, Lyman & Lackey.

MEDICAL REPORTER, a semi-monthly record of medicine and surgery, edited by Drs. J. S. B. Alleyne and O. F. Potter, $3 a year; St. Louis, Mo.

PHRENOLOGICAL JOURNAL, $2 a year, 389 Broadway, New York, by Fowler and Wells.

